# Prevalence and spectrum of cancer predisposition germline mutations in young patients with the common late‐onset cancers

**DOI:** 10.1002/cam4.6445

**Published:** 2023-08-23

**Authors:** Shaoyu Hao, Ximeng Zhao, Yue Fan, Zhengchuang Liu, Xiang Zhang, Wei Li, Hongling Yuan, Jie Zhang, Yanxiang Zhang, Tonghui Ma, Houquan Tao

**Affiliations:** ^1^ Thoracic Surgery, Shandong Cancer Hospital and Institute, Shandong First Medical University and Shandong Academy of Medical Sciences Jinan China; ^2^ Jichenjunchuang Clinical Laboratory Hangzhou China; ^3^ Department of Integrated Traditional Chinese Medicine and Western Medicine Zhong Shan Hospital, Fudan University Shanghai China; ^4^ Key Laboratory of Gastroenterology of Zhejiang Province Zhejiang Provincial People's Hospital, People's Hospital of Hangzhou Medical College Hangzhou China; ^5^ Department of Gastroenterology Zhejiang Provincial People's Hospital, People's Hospital of Hangzhou Medical College Hangzhou China

**Keywords:** cancer, Chinese, early onset, germline variation

## Abstract

**Background:**

Pathogenic germline variants (PGVs) can play a vital role in the oncogenesis process in carriers. Previous studies have recognized that PGVs contribute to early onset of tumorigenesis in certain cancer types, for example, colorectal cancer and breast cancer. However, the reported prevalence data of cancer‐associated PGVs were highly inconsistent due to nonuniform patient cohorts, sequencing methods, and prominent difficulties in pathogenicity interpretation of variants. In addition to the above difficulties, due to the rarity of cases, the prevalence of cancer PGV carriers in young cancer patients affected by late‐onset cancer types has not been comprehensively evaluated to date.

**Methods:**

A total of 131 young cancer patients (1–29 years old at diagnosis) were enrolled in this study. The patients were affected by six common late‐onset cancer types, namely, lung cancer, liver cancer, colorectal cancer, gastric cancer, renal cancer, and head–neck cancer. Cancer PGVs were identified and analyzed. based on NGS‐based targeted sequencing followed by bioinformatic screening and strict further evaluations of variant pathogenicity.

**Results:**

Twenty‐three cancer PGVs in 21 patients were identified, resulting in an overall PGV prevalence of 16.0% across the six included cancer types, which was approximately double the prevalence reported in a previous pancancer study. Nine of the 23 PGVs are novel, thus expanding the cancer PGV spectrum. Seven of the 23 (30.4%) PGVs are potential therapeutic targets of olaparib, with potential implications for clinical manipulation. Additionally, a small prevalence of somatic mutations of some classic cancer hallmark genes in young patients, in contrast to all‐age patients, was revealed.

**Conclusion:**

This study demonstrates the high prevalence of PGVs in young cancer patients with the common late‐onset cancers and the potentially significant clinical implications of cancer PGVs, the findings highlight the value of PGV screening in young patients across lung cancer, liver cancer, colorectal cancer, gastric cancer, renal cancer, or head–neck cancer.

## 1 INTRODUCTION

Due to decreasing costs and popularization of high‐throughput sequencing, studies of cancer genetics were extensively conducted during the last decade. It has become clear that pathogenic germline variants (PGVs) in cancer predisposition genes play a vital role in cancer susceptibility and aggressiveness,^1^ and significantly affect clinical manipulations.^2^ For example, a recent large‐cohort study across more than 50 malignancies reported that 17% of advanced cancer patients harbor cancer‐associated PGVs, and 9% of whom had therapeutically actionable PGVs according to OncoKB classification.^3^ However, the overall prevalence of cancer PGVs has been inconsistent, as a result of combined influences, including nonuniform patient inclusion criteria, sequencing methods, and variant pathogenicity interpretation protocols in different studies. Indeed, the reported prevalence of cancer PGVs vary widely from 4.3% to 19.7%.^4^, ^5^, ^6^ Despite the inconsistent prevalence of PGVs, these studies highlight the importance of germline variation screening in both cancer patients and their relatives. Germline variation screening may help in early diagnosis of hereditary cancer, with the promise of therapeutic benefit.

Special attention should be given to the fact that cancer incidence among youth cases has been consistently increasing in the recent decades.^7^ The increase in germline variation rates underlying unknown causes is a potential contributing factor.^8^, ^9^ Previous pancancer analyses have reported a PGV prevalence of 8% among the all‐age TCGA cohort^6^ and 8.5% among pediatric cancer patients (<20 years of age),^10^ revealing no obvious difference between the two groups. However, according to studies conducted for single cancer types (e.g., lung adenocarcinoma, colon cancer, breast cancer, and renal cancer), PGV carriers were found to be prone to develop cancers at younger ages,^11^, ^12^, ^13^, ^14^ with contradictory results to pancancer research. This is likely due to the inclusion of cancers that are intrinsically prone to affect young people in pancancer studies, for example, central nervous system tumors, hematological tumors, and sarcomas. These results indicate that despite intensively conducted studies, the prevalence and spectrum of cancer PGVs as well as their associated oncogenic mechanisms have not been thoroughly evaluated, especially for young patients who are affected by common late‐onset cancer types due to their rarity. Therefore, it is necessary to perform studies in large and pancancer cohorts to comprehensively evaluate the role of PGVs on accelerating cancer onset.

In this study, we retrospectively enrolled 131 young Chinese cancer patients (1–29 years old) diagnosed with the common late‐onset cancer types including lung cancer, liver cancer, colorectal cancer, gastric cancer, renal cancer, and head–neck cancer. NGS‐based targeted sequencing was performed to identify cancer‐associated PGVs and describe their contribution to early‐onset cancer. Somatic mutation spectra of cancers were compared between young PGV and non‐PGV carriers, as well as between early‐onset and all‐age cancers from cBioPortal database, to explore cancer somatic mutation characteristics associated with PGV predisposition.

## 2 RESULTS

### 2.1 Prevalence of young patients with PGVs in common late‐onset cancer types

**FIGURE 1:**
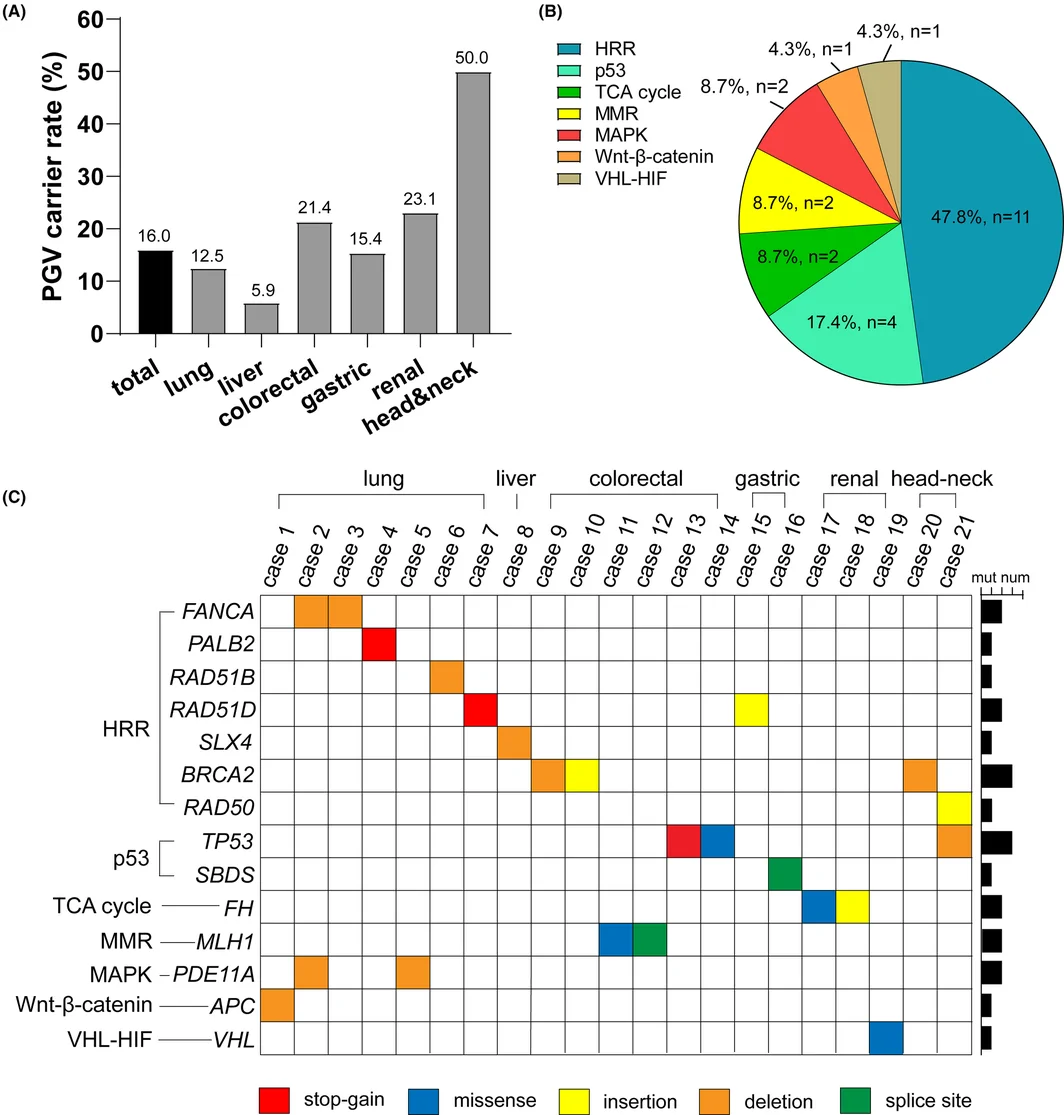
(A) PGV prevalence, overall and in each cancer type. (B) Proportions of pathways in which germline variant genes are involved. (C) Heatmap showing that PGVs are enriched in different pathways in each cancer type. Variant types are distinguished by color, and columns on the right side indicate the number of PGVs in each gene. PGV, pathogenic germline variants.

A total of 131 young patients were enrolled in this study. During the pathogenicity evaluation process, 23 PGVs in 21 patients were identified (Table 1). Among the 21 PGV carriers, patient no.2 and no.21 carried two PGVs, respectively. Eighteen of 21 genomic DNA (gDNA) samples from the PGV carriers were available, and Sanger sequencing was performed and successfully validated 19 of 23 PGVs. For the four variants which were not validated by Sanger sequencing due to the lack of three gDNA samples and difficulty in PCR amplification at one variant site, the mutational sites shown with Integrative Genomics Viewer (IGV) are provided (Figure S1). The average age at diagnosis of PGV carriers was 25.3 (range, 16–29) years, that is, no PGV was identified in children (1–12 years old) in the enrolled young patients. Notably, PGV carriers accounted for as much as 16.0% (21/131) of all young patients across six common late‐onset cancer types, the prevalence was found 12.5% (7/56) for lung cancer, 5.9% (1/17) for liver cancer, 21.4% (6/28) for colorectal cancer, 15.4% (2/13) for gastric cancer, 23.1% (3/13) for renal cancer, and 50.0% (2/4) for head–neck cancer (Figure 1A).

**TABLE 1:** Detailed information of identified PGVs and carriers.

Patient no.	Cancer type	Age at diagnosis	Gender	Germline variant gene	cDNA change	Protein change	mRNA refseq	VAF	Novel variant	Novel correlation	Pathway
1	Lung	29	M	*APC*	c.3183_3187del	p.Gln1062*	NM_000038.5	47.1%	N	N	Wnt‐β‐catenin
2	Lung	24	F	*FANCA*	c.3520_3522del	p.Trp1174del	NM_000135.2	43.6%	N	N	HRR
				*PDE11A*	c.20_21del	p.Asp7Valfs*16	NM_016953.3	48.0%	Y	Y	MAPK
3	Lung	28	F	*FANCA*	c.987_990del	p.His330Alafs*4	NM_000135.2	41.3%	N	N	HRR
4	Lung	16	M	*PALB2* ^a^	c.613G>T	p.Glu205*	NM_024675.3	47.3%	Y	Y	HRR
5	Lung	29	F	*PDE11A*	c.20_21del	p.Asp7Valfs*16	NM_016953.3	48.0%	Y	Y	MAPK
6	Lung	22	F	*RAD51B* ^a^	c.117_130del	p.Met39Ilefs*8	NM_133509.3	32.5%	Y	N	HRR
7	Lung	27	F	*RAD51D* ^a^	c.817C>T	p.Arg273*	NM_001142571.1	49.1%	Y	N	HRR
8	Liver	23	F	*SLX4*	c.2808_2809del	p.Ala938Thrfs*7	NM_032444.2	53.3%	N	Y	HRR
9	Colorectal	27	F	*BRCA2* ^a^	c.5993_5994del	p.Gln1998Argfs*4	NM_000059.3	44.8%	N	N	HRR
10	Colorectal	24	M	*BRCA2* ^a^	c.4276dup	p.Thr1426Asnfs*12	NM_000059.3	47.9%	Y	N	HRR
11	Colorectal	29	M	*MLH1*	c.544A>G	p.Arg182Gly	NM_000249.3	39.5%	N	N	MMR
12	Colorectal	29	M	*MLH1*	c.790+3A>T	Undetermined	NM_000249.3	41.7%	N	N	MMR
13	Colorectal	29	M	*TP53*	c.916C>T	p.Arg306*	NM_001126112.2	49.5%	N	N	p53
14	Colorectal	18	F	*TP53*	c.451C>A	p.Pro151Thr	NM_001126112.2	48.7%	N	N	p53
15	Gastric	27	M	*RAD51D* ^a^	c.330_331dup	p.Lys111Ilefs*13	NM_001142571.1	51.3%	Y	N	HRR
16	Gastric	20	F	*SBDS*	c.258+2T>C	p.Cys84fs*3	NM_016038.2	36.0%	N	N	p53
17	Renal	27	M	*FH*	c.698G>A	p.Arg233His	NM_000143.3	46.3%	N	N	TCA cycle
18	Renal	24	M	*FH*	c.1394_1395dup	p.Asp466Metfs*18	NM_000143.3	54.3%	Y	N	TCA cycle
19	Renal	26	M	*VHL*	c.269A>T	p.Asn90Ile	NM_000551.3	48.0%	N	N	VHL‐HIF
20	Head–neck	25	F	*BRCA2* ^a^	c.5414del	p.Asn1805Metfs*10	NM_000059.3	48.2%	Y	N	HRR
21	Head–neck	29	M	*RAD50*	c.2165dup	p.Glu723Glyfs*5	NM_005732.3	25.6%	N	N	HRR
				*TP53*	c.994del	p.Ile332Serfs*13	NM_001126112.2	44.5%	Y	N	p53

Abbreviations: B, benign; D, damaging; F, female; HRR, homologous recombination repair; M, male; N, no; PGV, pathogenic germline variants; PoD, possibly damaging; PrD, probably damaging; T, tolerated; VAF, variant allele frequency; Y, yes.

*Note*: The protein change caused by the intron single nucleotide substitution in patient 17 was validated in a previous study.^15^

^a^ In the germline variant gene column indicates potentially targetable genes.

### 2.2 Spectrum and characteristics of PGVs carried by young patients

Totally, 23 PGVs in 14 unique genes were identified in this study: *APC*, *BRCA2*, *FANCA*, *FH*, *MLH1*, *PALB2*, *PDE11A*, *RAD50*, *RAD51B*, *RAD51D*, *SBDS*, *SLX4*, *TP53*, and *VHL*. Detailed information on the PGV carriers and PGVs is listed in Table 1. According to Tumor Suppressor Gene Database, all of the above genes were tumor suppressors, except for *SBDS*, as its cancer‐associating mechanism is not fully understood. Most identified variants are predicted to be truncating (*n* = 17, 73.9%), including three nonsense single nucleotide variations (SNVs), one splice site SNV and 13 small indels predicted to cause frame‐shift and premature termination during translation, and will probably cause decisive damage to gene functions, while four missense variants (17.3%), one in‐frame deletion (4.3%) and one splice site mutation (4.3%) with undetermined protein change, were identified. The variant genes were then classified based on their related cancer pathways. Overall, homologous recombination repair (HRR) (*n* = 11, 47.8%) and p53 (*n* = 4, 17.4%) were the top two enriched pathways (Figure 1B). Consistent with the results, the gene ontology (GO) enrichment analysis also showed that double‐strand break repair is the most enriched GO pathway for the variant genes (Figure S2). Among the different cancer types, the variant genes were enriched in distinctive cancer pathways (Figure 1C). This was observed predominantly in lung cancer and renal cancer, for which the most frequent variant genes are involved in the HRR (*n* = 5, 62.5%) and TCA cycle (*n* = 2, 66.7%) pathways, respectively. Among the identified PGVs, seven (7/23, 30.4%) are in four HRR genes: *PALB2*, *RAD51B*, *RAD51D*, and *BRCA2*. These variants may be targeted by the PARP inhibitor olaparib. According to the OncoKB database.^2^

### 2.3 Novel PGV identification

Nine novel PGVs were identified (Table 1), expanding the PGV spectrum for young cancer patients. The novel PGVs are exclusively truncating variants in tumor suppressor genes. These truncating variants presumably lead to loss‐of‐function (LOF) of tumor suppressors, resulting in oncogenesis. In addition, three novel correlations were indicated in this study, namely the correlation of *PDE11A* and *PALB2* with lung cancer, and that of *SLX4* with liver cancer, while the rest of genes identified in this study have known correlations with their corresponding cancer types (Table 1) according to Human Gene Mutation Database (HGMD) and previous studies.^6^, ^16^, ^17^, ^18^, ^19^, ^20^, ^21^, ^22^ All variants with novel correlations with their respective cancer types are also truncating. The pathogenicity of novel PGVs and reliability of the novel correlations are supported by the rarity of early‐onset cancers and the predictively significant changes in cancer‐related protein functions caused by truncating PGVs.

### 2.4 Comparisons of somatic mutations between PGV and non‐PGV carriers

It is hypothesized that cancers developing in young PGV carriers may carry few somatic mutations. This is because both PGVs and somatic mutations are involved in oncogenesis and because involvement of PGVs may lower the threshold for the accumulation of somatic mutations needed for disease onset.

**FIGURE 2:**
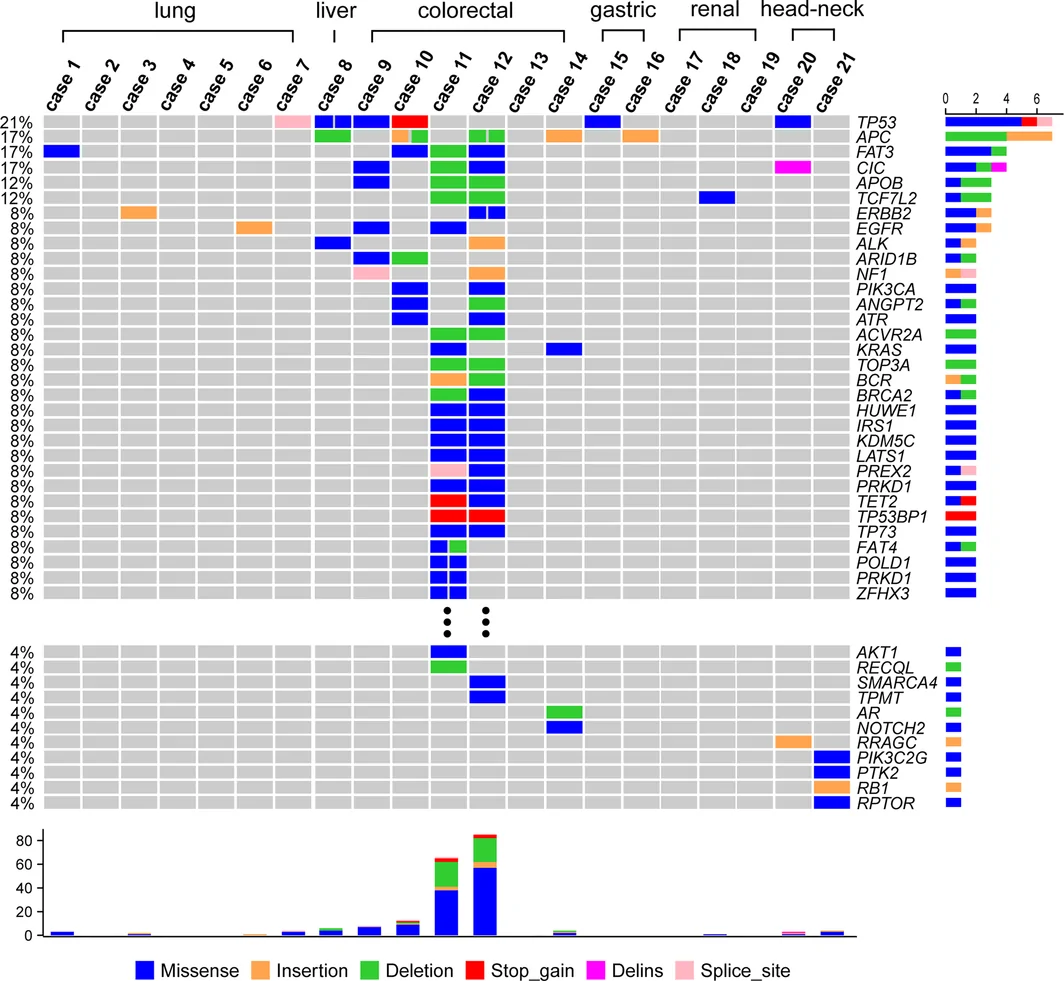
Spectrum of somatic mutations in PGV‐carrier tumor samples. The number of mutations is shown as columns at the right side and the bottom. Mutation types are distinguished by color. For cases 11 and 12 carrying *MLH1* PGVs, some somatic mutations are not shown in the heatmap due to their large quantity. PGV, pathogenic germline variants.

To test this hypothesis, tumor mutation burden (TMB) was compared between PGV carriers and non‐PGV carriers across six cancer types. However, no significant differences in TMBs were identified (*p* = 0.32 for lung cancer; *p* = 0.26 for non‐*MLH1*‐mutated colorectal cancer; *p* = 0.68 for gastric cancer; *p* = 0.42 for renal cancer; *p* = 0.99 for head–neck cancer; and *p* = 0.08 for overall cancers). For liver cancer, this statistic was not applicable because the Mann–Whitney test needs at least two values for each group. The somatic variants identified in the PGV carriers are illustrated in Figure 2 and listed in Table S1. Notably, the comparison of overall cancers with/without PGV carriers showed the lowest p value, which indicated that the insignificant statistical results may be due to the small sample size. The number of driver mutations (identified based on the driver mutation database InTOgen^23^) between PGV carriers and non‐PGV carriers in each cancer type was also evaluated, but no statistical significance was observed.

For PGV carriers, the second hit in a wild‐type allele in tumor was not detected for the genes evaluated. This suggests that the two‐hit event is an uncommon driver for oncogenesis in young PGV carriers. Two of the colorectal cancers carrying *MLH1* PGVs had the highest TMB (cases 11 and 12), due to disrupted DNA mismatch repair functions. For all cancer types, the most frequently mutated gene was *TP53* (*n* = 7) and *APC* (*n* = 7), followed by *FAT3* (*n* = 4) and *CIC* (*n* = 4), *APOB* (*n* = 3), and *TCF7L2* (*n* = 3).

### 2.5 Different hallmark somatic mutation frequencies between early‐onset and all‐age cancers

To explore whether differences in somatic hallmark gene mutation frequencies between early‐onset and all‐age cancers exist, a dataset of all‐age Chinese cancer patient somatic mutation data published at cBioPortal were examined in this study.^24^ For each cancer type, the top three mutated genes in the COSMIC database were analyzed, and significant differences in mutation carrier rates in lung cancer, colorectal cancer, and renal cancer were identified (Table 2). These results indicate that the oncogenic basis for early‐onset cancers is disparate for some specific cancer types. Notably, mutated EGFR in lung cancer and KRAS in colorectal cancer are therapeutic targets of FDA‐approved drugs, and their lower carrier rates in early‐onset cancers may restrict relevant drug applications.

**TABLE 2:** Comparisons of somatic mutation numbers in cancer hallmark genes between early‐onset and all‐age cancers.

Cancer type	Mutation gene	Somatic mutation carrier rate (early‐onset vs. all‐age)	Corrected *χ* ^2^	*p*‐Value
Lung	*EGFR* ^a^	12.5% vs. 47.0%	24.72	**<0.0001** ***
	*TP53*	30.4% vs. 57.1%	14.83	**<0.001** **
	*LRP1B*	5.4% vs. 18.1%	5.25	**0.02** *
Liver	*TP53*	61.1% vs. 58.0%	0.00	0.98
	*LRP1B*	0.0% vs. 12.6%	1.56	0.21
	*PREX2*	0.6% vs. 6.6%	0.09	0.77
Colorectal	*APC*	46.4% vs. 70.0%	6.08	**0.01** *
	*TP53*	71.4% vs. 74.1%	0.01	0.92
	*KRAS* ^a^	25.0% vs. 48.2%	5.00	**0.03** *
Gastric	*TP53*	53.8% vs. 61.5%	0.08	0.78
	*LRP1B*	0.0% vs. 19.5%	2.01	0.16
	*FAT4*	15.4% vs. 14.4%	0.09	0.76
Renal	*VHL*	0.0% vs. 57.5%	16.82	**<0.0001** ***
	*PBRM1*	0.0% vs. 30.5%	5.34	**0.02** *
	*SETD2*	0.0% vs. 14.9%	1.53	0.22
Head–neck	*TP53*	25% vs. 22.9%	0.25	0.62
	*NOTCH1*	0.0% vs. 6.9%	0.22	0.64
	*FAT1*	25% vs. 4.6%	0.48	0.49

The bold font indicates *p*‐values with statistical significance. “*”; “**” and “***” indicate *p*‐values smaller than 0.05; 0.001 and 0.0001, respectively.

^a^ represents potentially targetable genes

## 3 DISCUSSION

This study aimed to evaluate PGVs in young patients with the common late‐onset cancers. In this study, we included children (1–12 years old), adolescents (13–17 years old), and young adults with restricted age range (18–29 years old). To reveal the relationship between cancer predisposition germline mutations and “young patients”, 30–39 year olds were excluded in the present study. This is because the incidences of each included cancer type increase notably in patients aged 30–39 comparing to those who were aged 20–29, and reach the highest peak in the range of >70 years old, according to the Surveillance, Epidemiology, and End Results (SEER) data (https://seer.cancer.gov/statistics‐network/). Moreover, the previous studies focusing on early‐onset cancer, flexible age ranges were applied, for example, 0–20 years old or 0–25 years old.^10^, ^25^ Our results showed that as many as 16.0% of young patients carried one or two PGVs, which were approximately double that of previous pancancer PGV studies for both adult and pediatric cohorts.^6^, ^10^ These differences may have been caused by a series of factors, such as the ethnicity of the patients, the differing age thresholds for the definition of “young” patient, the differing constitution of included cancer types, the differing gene sets under evaluation, and the inconsistent standards for classifying pathogenicity of variants. We applied a comprehensive and strict workflow to evaluate the pathogenicity of germline variants. This stringent evaluation standard led to a remarkably high identification rate of truncating variants, at 73.9%. Considering the stringency of our evaluation and that the pathogenicity of many missense variants classified “VUS” was difficult to confirm, it is rational to speculate that the actual prevalence of PGVs in young patients is even higher. Therefore, to some extent, the strict evaluation of variant pathogenicity might be regarded as a limitation of this research. Regarding clinical practice, these results strongly suggest that young patients affected by common late‐onset cancer types, as well as their risk‐relevant relatives, should undergo germline variant cascade screening, regardless of whether they have clarified family histories.

Nine novel variants were identified in this study, and eight of nine novel variants identified are truncating variants in tumor suppressor genes. Speculatively, these variants would lead to silencing of mutated alleles and haploinsufficiency. The function of *SBDS* in cancer is still unclear^26^; however, with respect to evidence for its tumor suppressor function, an *SBDS* germline stop‐gain variant has previously been identified in gastric cancer.^20^ Here, we provide additional evidence supporting its tumor suppressor function; that is, an *SBDS* germline truncating variant was detected in a 20‐year‐old gastric cancer patient, and no other PGV in the same patient was identified.

Three novel gene–cancer associations were found in this study, namely, *PDE11A* and *PALB2* with lung cancer and *SLX4* with liver cancer. LOF variants in *PDE11A* have been reported to cause testicular germ cell tumor^27^ and prostate cancer^28^ through increased cAMP levels. According to another in vitro study, elevation of cAMP can delay nonhomologous end joining DSB repair in lung cancer cells.^29^ This suggests that *PDE11A* has a tumor‐suppressing function in the lungs. In this study, the recurrent *PDE11A* c.20_21del truncating variants were found in two lung cancer patients, suggesting its correlation with lung cancer and tumor suppression. Additionally, the *PALB2* c.613G>T germline variant, causing premature termination of translation, was identified in one patient (number 2). *PALB2* is a thoroughly studied predisposition gene in breast cancer, and almost all truncating variants are damaging. *PALB2* has also been associated with ovarian and pancreatic cancer; however, our study is the first report of *PALB2* PGVs in lung cancer, and further statistical genetic evidence and functional studies are needed to support this association. Additionally, one *SLX4* PGV was identified in liver cancer. Involvement of *SLX4* germline variants in oncogenesis is still debatable. As supportive evidence, *SLX4* PGV carriers among high‐grade serous ovarian cancer patients have an odd ratio of 4.07,^30^ and the *SLX4* p.A938Tfs*7 germline variant has been previously identified in prostate cancer.^31^

According to a previous study, cancer‐affiliated germline variant burden correlates inversely with cancer onset age and somatic mutation burden,^32^ which is consistent with our results showing that young patients have a remarkably high PGV prevalence. However, the suspected lower somatic mutation number in young PGV carriers was not observed when compared with non‐PGV carriers. This was possibly due to the limited sample size. Previous functional studies indicated that early onset of cancer is associated with synergy effect of germline variants and somatic variants,^33^, ^34^ this is in accordance with our findings that oncogenesis at young age can be triggered by DNA impairment underlying PGVs, and the cancer proliferation is more related to somatic variants enriched in gland development, histone modification and epithelial cell proliferation pathways (Figure S2). However, to adequately evaluate the association between age, germline variants, and somatic variants, further large‐scale studies are needed. Additionally, our results showed that differences in hallmark somatic mutations exist between early‐onset and all‐age cohorts in lung, colorectal, and renal cancers. Importantly, lower mutation rates in two FDA‐approved therapeutic targets, namely, *EGFR* in lung cancer and *KRAS* in colorectal cancer, were identified, indicating that young patients with lung cancer or colorectal cancer may have a smaller chance of benefitting from targeted therapies. The difference in carrier rates of *EGFR* mutations between early‐onset and late‐onset lung cancer cohorts is highly debatable according to a previous study,^35^ though the lower *KRAS* mutation carrier rate in early‐onset colorectal cancer is consistent with one previous report.^36^ Despite the lower chance of utilizing somatic mutations as therapeutic targets in some early‐onset cancers, we found that as many as 30.4% of PGVs are potential targets of olaparib for the included cancer types among early‐onset patients. olaparib has been approved by the USFDA for treating prostate cancer harboring LOF variants of the above four genes. Nevertheless, these anticipated therapeutic effects should be evaluated in clinical studies.

The above conclusions further increase the value of PGV screening in young cancer patients.

## 4 CONCLUSIONS

Under strict evaluation criteria for annotating variant pathogenicity, this research identified high PGV prevalence among young patients with the common late‐onset cancers and novel gene–cancer associations. The results indicated that cancer PGVs are associated not only with cancer aggressiveness, but also with early onset. Notably, nearly one third of the PGVs identified in the study are potentially targetable. This conclusion calls for further functional and clinical studies to validate their therapeutic implications. Additionally, the results strongly indicate the importance of PGV screenings in young cancer patients, which would contribute to early diagnosis and prevention of hereditary cancers and identify novel therapeutic targets to support clinical manipulations.

## 5 MATERIALS AND METHODS

### 5.1 Enrollment of young patients and collection of specimens

To evaluate PGVs in early‐onset patients, children (1–12 years), adolescents (13–17 years), and young adults with restricted age range (18–29 years) diagnosed with lung, liver, colorectal, gastric, renal, or head–neck cancer, which are common late‐onset cancer types included. The histopathology information for each patient is provided in Table S1. To strictly distinguish early‐onset patients from others and meet the aim of this study, we adopted a strict upper‐limit age for the inclusion of AYA patients. According to the SEER database, the highest age at diagnostic brackets of our included cancer types in non‐Hispanic Asian/pacific islander were exclusively over 70 years. Furthermore, patients diagnosed with embryonal malignancies that are susceptible to children were excluded by pathological results. Patients were initially diagnosed in Shandong Cancer Hospital, Zhong Shan Hospital, or Zhejiang Provincial People's Hospital between 2019 and 2022. Matching tumor tissue and peripheral blood sample pairs from each patient were collected and preserved for subsequent testing. This study was approved by the ethics committee of Zhejiang Provincial People's Hospital (QT2022087). All patients provided informed consent to participate in this study.

### 5.2 Panel sequencing, variant calling, and evaluation of pathogenic variants

gDNA was extracted from each tumor tissue sample and its matched white blood cells as genetic background using the QIAamp DNA Mini Kit (QIAGEN) and DNA Blood Midi/Mini kit (QIAGEN), respectively. DNA fragmentation, library construction, and sequencing were described in a previous study.^37^ Briefly, gDNA were sheared in ~200 bp fragments by M220 Focused‐ultrasonicator (Covaris). DNA libraries were constructed with KAPA HTP Library Preparation Kit (KAPA Biosystems), the libraries were captured by 825‐gene panel designed for detecting somatic and germline variants in cancer‐related genes, and subsequently sequenced with the Novaseq platform. The sequencing depth of the target regions is more than 500x per tumor tissue sample and 100× per control.

Raw data were processed by Trimmomatic (Ver 0.36) and FastQC (version 0.11.2), and aligned to the reference genome (GRch37) using Burrows‐Wheeler Aligner tool (BWA, v0.7.10‐r789). Somatic SNVs were called using muTech (v3.1‐0‐g72492bb) and small insertion/deletions (indels) were called by Strelka (v1.0.14). Germline SNVs and small Indels were called using GATK (v3.1‐0‐g72492bb). Annotation of variants was performed using VEP (v83) and Oncotator (v1.5.1.0).^38^, ^39^ After annotation, the retained non‐synonymous SNVs and small indels were screened with variant allele frequency (VAF) (cutoff ≥5%) for tumor tissue sample and were filtered with VAF (cutoff ≥20%) for genetic background.

**FIGURE 3:**
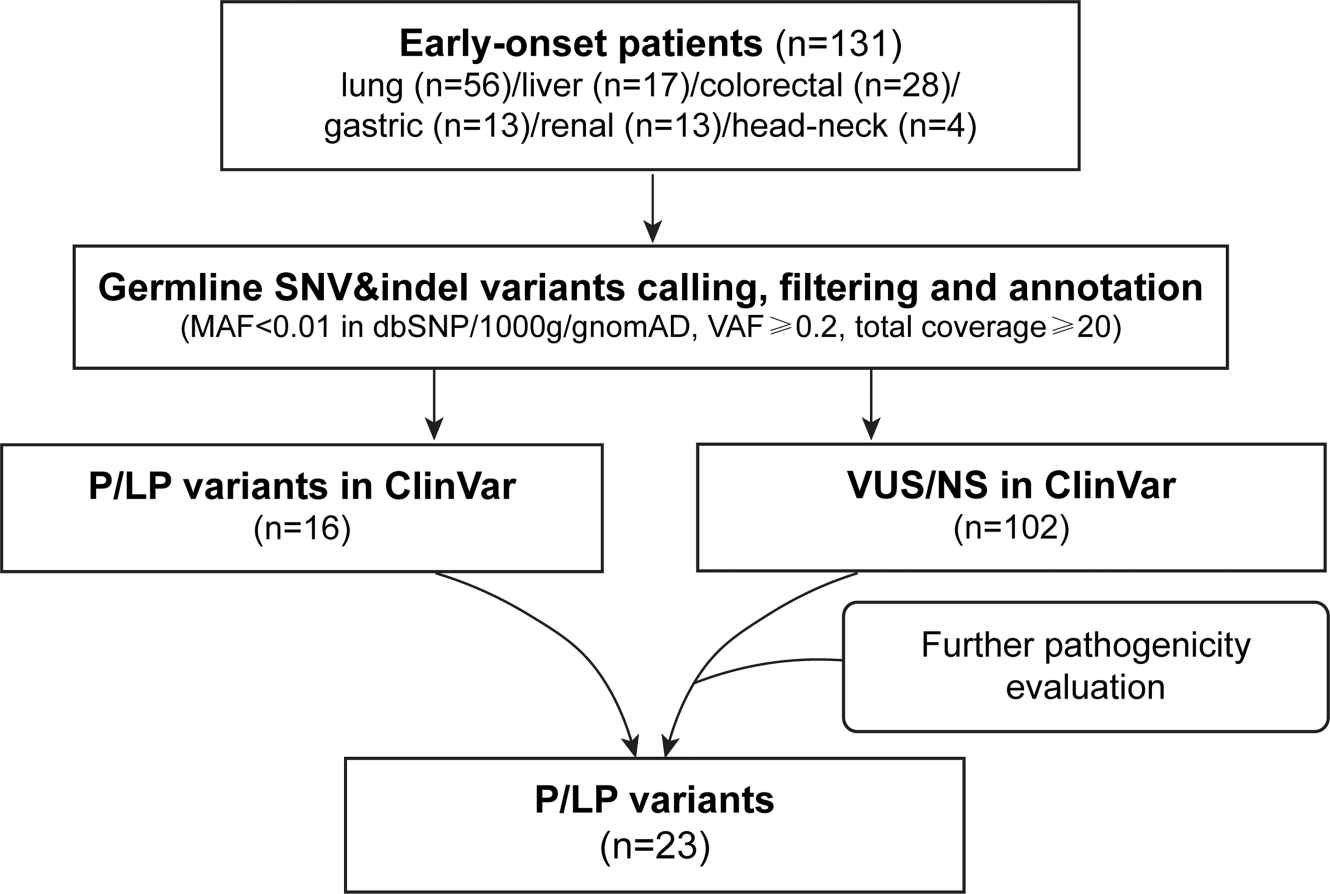
Workflow diagram for PGV identification. “*n*” represents the patient number in the “early‐onset patients” box and the candidate variant number in the other boxes. The detailed methods to identify P/LP variants are explained in the Section 5. LP, likely pathogenic; NS, not specified; P, pathogenic; PGV, pathogenic germline variants; SNV, single‐nucleotide variant; VAF, variant allele frequency; VUS, variants of unknown significance.

The germline variants were comprehensively reviewed and evaluated to identify “pathogenic” (P) and “likely pathogenic” (LP) SNVs and indels, based on their rarity (minor allele frequency, MAF <0.01) in population frequency databases (dbSNP, 1000 Genomes Project, and gnomAD), variant function computationally predicted by SIFT and PolyPhen2 (Table S2), and phenotype or disease databases (OMIM and ClinVar) were used to identify known P and LP variants. All variants annotated as “VUS” and not specified (NS) variants were then further evaluated manually to identify variants that are very likely to be deleterious. To clarify, truncating variants classified as “VUS” and NS in cancer‐suppressor genes located upstream of other ClinVar “P” or “LP” truncating variants were equally regarded as deleterious, as they also met the “P” or “LP” definition from Mendelics ClinVar Assertion Criteria (https://submit.ncbi.nlm.nih.gov/ft/byid/chhjzatu/mendelics_assertion_criteria_2017.pdf). Accordingly, all “VUS” and NS variants that did not meet the above criteria were excluded. As the final check, all the PGVs included meet the P/LP classification of American College of Medical Genetics and Genomics (ACMG) guidelines for interpretation of sequence variants. The determination of novelty for pathogenic variants and unknown associations between variant genes and specific cancer types was based on HGMD Professional 2022.1 and searched in PubMed. A brief pipeline for PGV identification is illustrated in Figure 3. The primers for PCR amplification and Sanger sequencing were designed by using Primer Premier 6. The bam files were visualized with IGV (version 2.11.9). GO enrichment analysis was performed with clusterProfiler (version 4.4.4). TMB was defined as the total number of somatic mutations per megabase of genome region examined and was calculated as the total number of nonsynonymous mutations.^40^

### 5.3 Statistical analyses

The chi‐squared test was applied to evaluate significant differences in PGV carrier numbers between sexes. The Mann–Whitney test was applied for comparations of two datasets (TMB and driver mutation numbers between PGV & non‐PGV carriers) that did not comply with a normal distribution. The chi‐squared test or Fisher's exact test was applied to evaluate differences in variant carrier rates between young and all‐age patients. The above analyses were performed using GraphPad Prism version 9.4.1, and *p* < 0.05 was considered statistically significant.

## AUTHOR CONTRIBUTIONS

**Shaoyu Hao:** Conceptualization (equal); data curation (lead); formal analysis (equal); investigation (equal); methodology (equal); resources (lead); writing – review and editing (equal). **Ximeng Zhao:** Formal analysis (lead); investigation (equal); methodology (equal); writing – original draft (lead); writing – review and editing (supporting). **Yue Fan:** Formal analysis (equal); investigation (equal); methodology (equal); resources (equal); writing – review and editing (equal). **Zhengchuang Liu:** Data curation (supporting); investigation (supporting); writing – review and editing (supporting). **Xiang Zhang:** Investigation (supporting); methodology (supporting). **Wei Li:** Project administration (lead). **Hongling Yuan:** Methodology (supporting); software (supporting). **Jie Zhang:** Project administration (supporting). **Yanxiang Zhang:** Conceptualization (equal); writing – review and editing (equal). **Tonghui Ma:** Conceptualization (equal); resources (equal); writing – review and editing (equal). **Houquan Tao:** Conceptualization (equal); data curation (equal); funding acquisition (lead); resources (lead).

## Supporting information


Figure S1:

Figure S2:

Table S1:

Table S2:

## Data Availability

The data that support the findings of this study are available from the corresponding author upon reasonable request.
