# Supplementary material for: Prevalence and spectrum of cancer predisposition germline mutations in young patients with the common late‐onset cancers
Source: Cancer Med. 2023 Aug 23;12(17):18394–404. doi: 10.1002/cam4.6445 (PMC10524041; doi:10.1002/cam4.6445)
Supplement: Supplementary file 1 — Figure S1: [file CAM4-12-18394-s004.pdf]

A

B

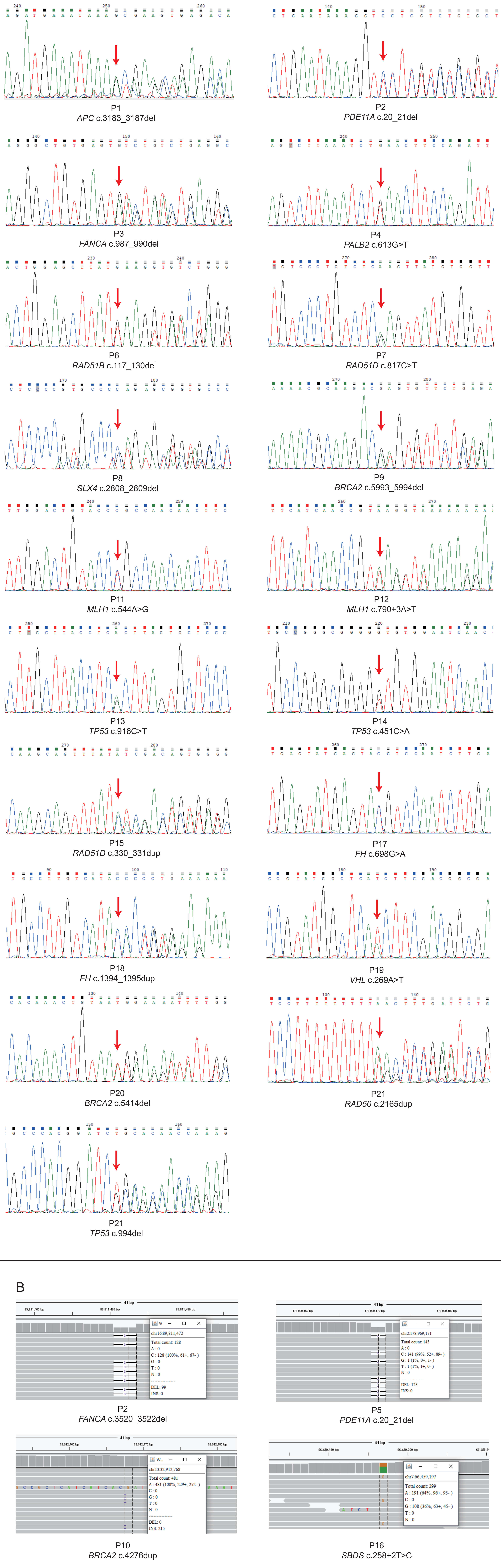

Supplementary Figure 1: Validations of germline variants. A, 19 of 23 variants were validated by Sanger sequencing, red arrows indicate the mutation sites. B, Screenshots from IGV software, showing the total count and count of variation reads at each site. “P” indicates patient number.
