# Supplementary material for: Prevalence and spectrum of cancer predisposition germline mutations in young patients with the common late‐onset cancers
Source: Cancer Med. 2023 Aug 23;12(17):18394–404. doi: 10.1002/cam4.6445 (PMC10524041; doi:10.1002/cam4.6445)
Supplement: Supplementary file 2 — Figure S2: [file CAM4-12-18394-s001.pdf]

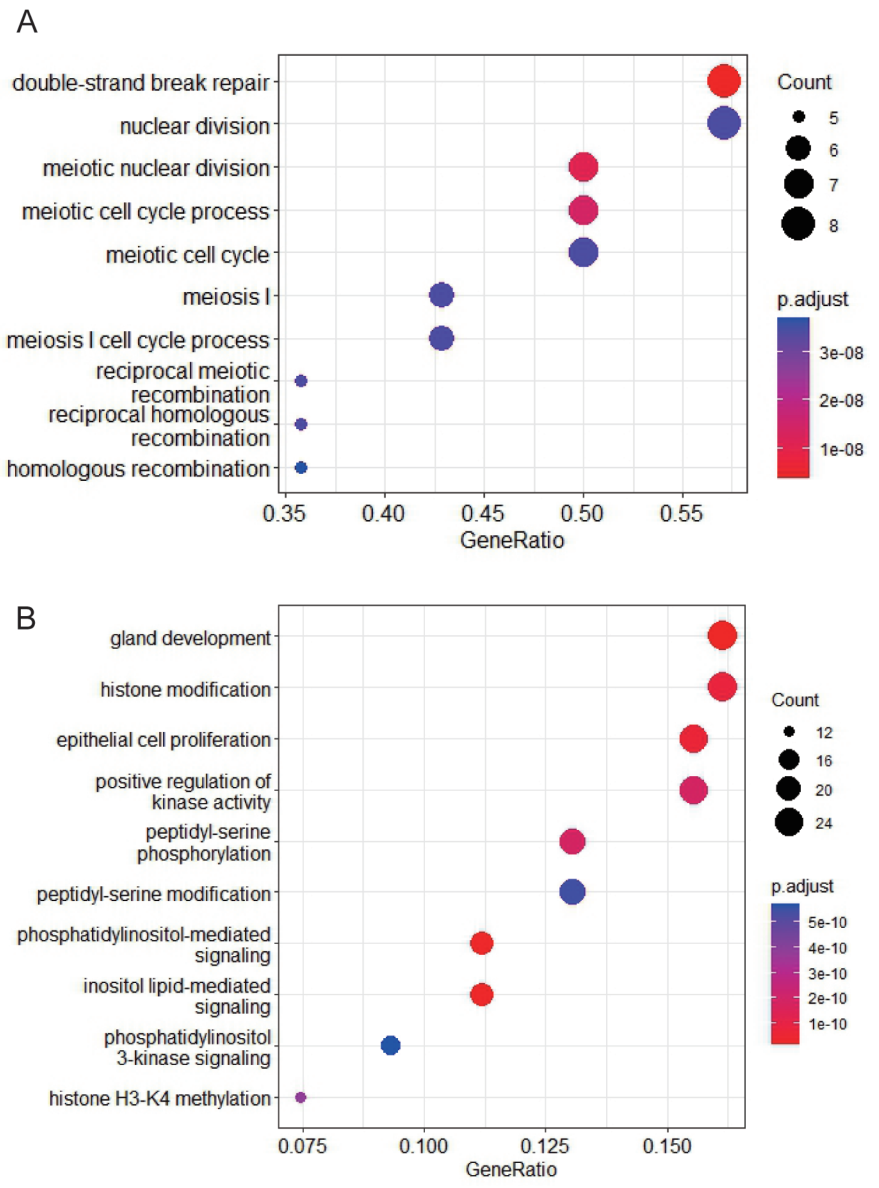

Supplementary Figure 2: GO pathway aggregation analysis of germline (A) and somatic (B) variants, showing top 10 enriched pathways respectively.
